# Supplementary material for: Defective Proinsulin Handling Modulates the MHC I Bound Peptidome and Activates the Inflammasome in β-Cells
Source: Biomedicines. 2022 Mar 30;10(4):814. doi: 10.3390/biomedicines10040814 (PMC9024965; doi:10.3390/biomedicines10040814)
Supplement: Supplementary file 1 [file biomedicines-10-00814-s001.zip › biomedicines-1611248-supplementary.pdf]

biomedicines

MDPI

Table S1. List of chemicals and antibodies used for experimental work related to this manuscript.

Reagent	Commercial vendor, Cat#
Ethanol	Ajax Finechem, cat. no. AJA214
Optima water	Thermo Fisher Scientific, cat. no. FSBW6-4
Methanol	Merck Millipore, cat. no. 1.06018.4000
Acetonitrile	Thermo Fisher Scientific, cat. no. FSBA955-4
Formic acid	Sigma-Aldrich, cat. no. 14265-1ML
Acetic acid	Sigma-Aldrich, cat. no. 33209-1L-GL
Igepal CA-630	Sigma-Aldrich, cat. no. I8896
Tris	Astral Scientific, cat. no. BIO3094T
**Antibodies**	
Anti-rabbit NLRP1	Cell signaling, cat. no. 4990S, dilution = 1:2,000
Anti-rat GRP94	Thermofisher Scientific, cat. no. MA3-016, dilution = 1:10,000
Anti-mouse proinsulin	Cell Signaling, cat. no. 8138S, dilution = 1:5000
Anti-mouse tubulin	Sigma, cat. no. T6074, dilution = 1:10,000
Anti-mouse IκB-α	Thermo Fisher scientific, cat. no. 40903, dilution = 1:1,000
Recombinant Anti-IL-1β	Abcam, cat. no. ab283818, dilution = 1:2,000
Anti-rat	Abcam, cat. no. ab6734, dilution = 1;10,000
Anti-rabbit	Cell signaling, cat. no. 7074S, dilution = 1;10,000
Anti-mouse	Cell signaling, cat. no. 7076S, dilution = 1;10,000

Table S2. List of peptides from heavy chain fraction digestion of INS-1E cells. The peptides map to RT1.A (class Ia) region of RT1 system in *Rattus norvegicus*.

Peptides obtained from tryptic digest of RT1.A heavy chain	Uniprot Accession for detected peptides	Protein description
**YDSDAENPR**	P15978|HA11_RAT	Class I histocompatibility antigen Non-RT1.A alpha-1 chain OS=*Rattus norvegicus* OX=10116 GN=RT1-Aw2 PE=1 SV=1
**WASVVVPLGK**	P15978|HA11_RAT:P16391|HA12_RAT	
**WEPSPSTDSNLLLLFLELWQFL**	P15978|HA11_RAT	
**GYEQHAYDGR**	P15978|HA11_RAT	
**DYIALNEDLK**	P15978|HA11_RAT:P16391|HA12_RAT	
**VEHEGLPEPLSQR**	P15978|HA11_RAT	RT1 class I histocompatibility antigen AA alpha chain OS=*Rattus norvegicus* OX=10116 PE=1 SV=2
**TWAVADFAAWITR**	P15978|HA11_RAT	

Table S3. List of RT1.A-bound peptides from individual replicates of INS-1E cells under tested conditions.

Table S4. List of peptides combined per condition for all groups.

Table S5. List of source proteins for RT1.A bound peptides for all tested groups.

Table S6. List of peptides with NNAlign predicted score and percentile rank for INS-1E cells under tested conditions.

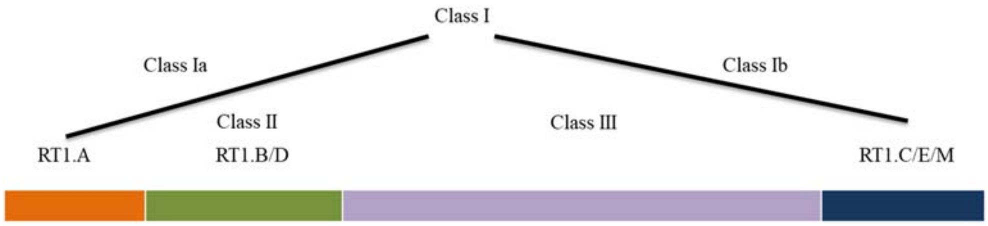

Figure S1. General genomic organization of RT1 system in rats. Localized on chromosome 20, the RT1 system has 4 main regions. The RT1.A is the class Ia region. The second region RT1.B/D includes class II genes. Class III contains non-MHC genes. Finally, the class Ib is represented by RT1.C/E/M.

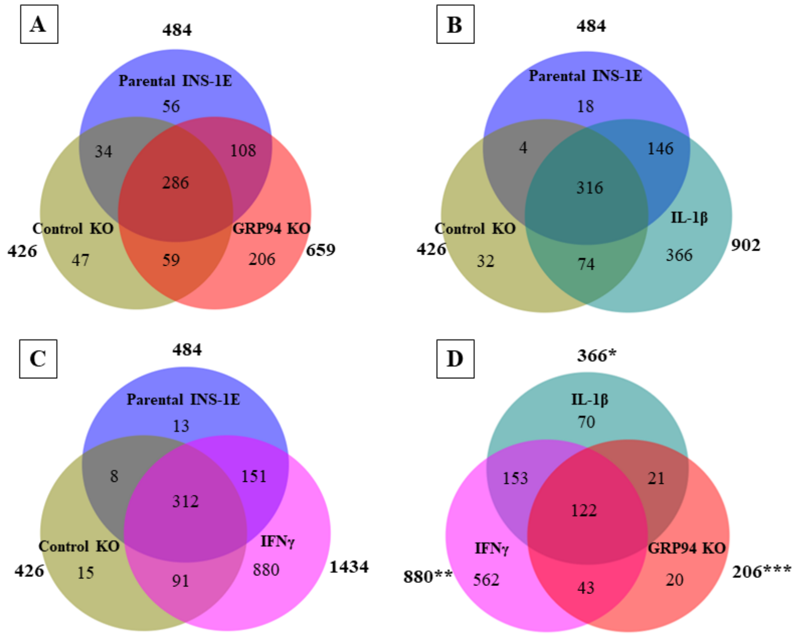

Figure S2. Venn diagrams showing number of source proteins for RT1.A eluted peptides distinct or overlapping between **(A)** Parental INS-1E, control KO & GRP94 KO and **(B)** Parental INS-1E, control KO & IL-1β exposure (15 pg/ml for 24 hours) **(C)** Parental INS-1E, control KO & IFNγ exposure (10 ng/ml for 24 hours) and **(D)** RT1.A contributing proteins exclusive for IL-1β, IFNγ exposed and GRP94 KO INS-1E groups. *n* = 2 for all except GRP94 KO where *n* = 4. * = IL-1β group proteins excluding parental INS-1E and control KO clone; ** = IFNγ group proteins excluding parental INS-1E and control KO clone; *** = GRP94 KO group proteins excluding parental INS-1E and control KO clone.

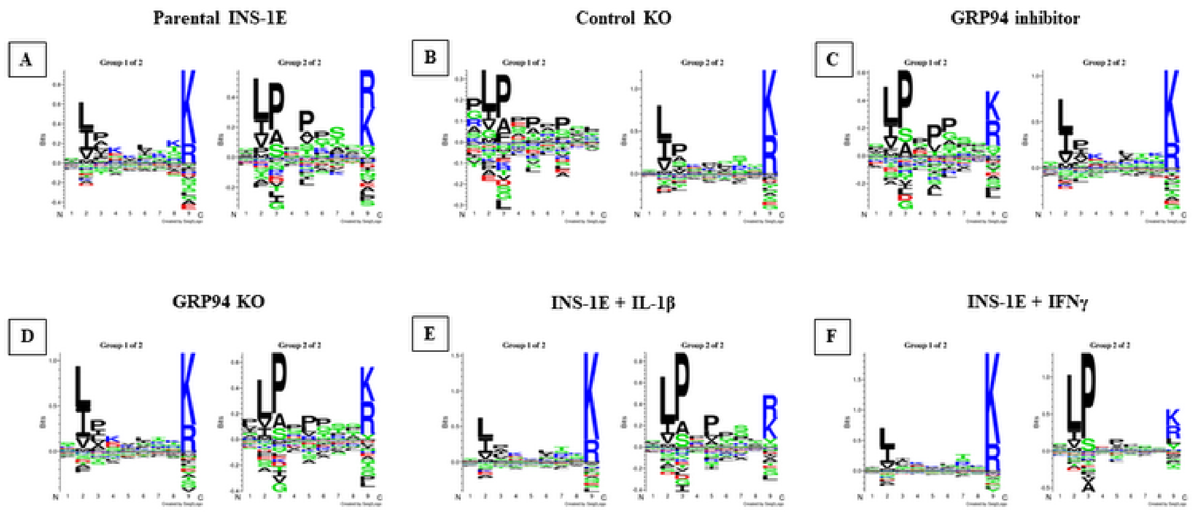

Figure S3. Gibbscluster analysis for RT1.A-bound peptides in INS-1E cells under tested conditions. **(A)** parental INS-1E **(B)** control GRP94 KO clone **(C)** INS-1E treated with GRP94 inhibitor (20 μM for 24 hours) **(D)** GRP94 KO INS-1E clone **(E)** IL-1β exposure (15 pg/ml for 24 hours) and **(F)** IFNγ exposure (10 ng/ml for 24 hours). *n* = 2 for all except GRP94 KO where *n* = 4.

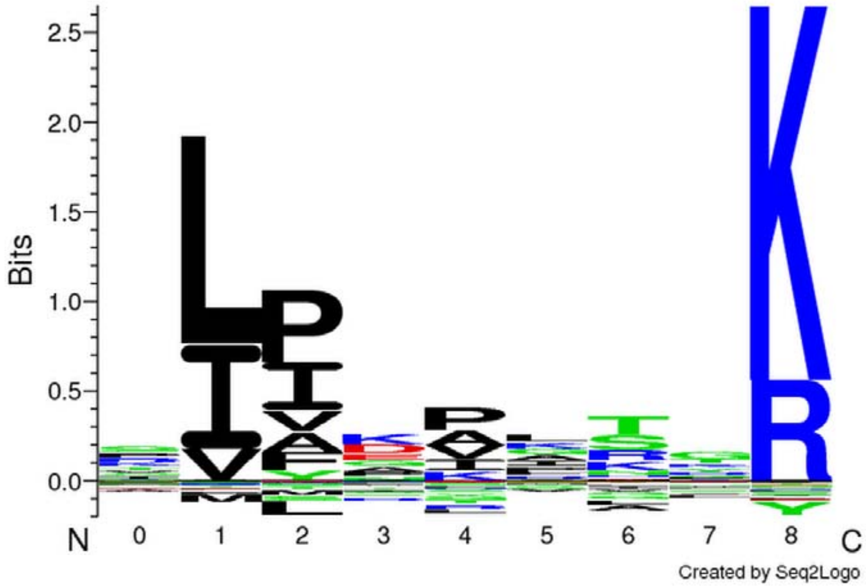

Figure S4. NNAlign binding motif for trained model against INS-1E peptides. The model was trained against RT1.A-bound and random natural peptides.
